# Supplementary material for: Hepatic stellate cells limit hepatocellular carcinoma progression through the orphan receptor endosialin
Source: EMBO Mol Med. 2017 Apr 3;9(6):741–9. doi: 10.15252/emmm.201607222 (PMC5452049; doi:10.15252/emmm.201607222)
Supplement: Supplementary file 1 — Appendix [file EMMM-9-741-s001.pdf]

Appendix

Hepatic stellate cells limit hepatocellular carcinoma progression through the orphan receptor endosialin

Carolin Mogler,[1,2,3]*, Courtney König[1,4]*, Matthias Wieland[1,4], Anja Runge[1,4], Eva Besemfelder[1], Dorde Komljenovic[5], Thomas Longerich[6], Peter Schirmacher[2], and Hellmut G. Augustin[1,4,7]

[1]Division of Vascular Oncology and Metastasis, German Cancer Research Center Heidelberg (DKFZ-ZMBH Alliance), Germany; [2]Institute of Pathology, Heidelberg University, Germany; [3]Institute of Pathology, Technical University Munich, Germany; [4]Department of Vascular Biology and Tumor Angiogenesis (CBTM), Medical Faculty Mannheim, Heidelberg University, Germany; [5]Department of Medical Physics in Radiology, German Cancer Research Center Heidelberg, Germany; [6]Institute of Pathology, RWTH Aachen, Germany; [7]German Cancer Consortium, Heidelberg Germany.

Table of contents

Appendix Figure S1: Endosialin expression on human dysplastic nodules (whole tissue slides).

Appendix Figure S2: Heterogeneous Endosialin expression on HCC tissue microarray punches.

Appendix Figure S3: Co-stainings of Endosialin and CD31 on human HCC.

Appendix Figure S4: Immunohistochemical analysis of non-tumorigenic WT and Endosialin-deficient livers.

Appendix Figure S5: Computer tomographical analysis of tumorigenic WT and Endosialin-deficient mice.

Appendix Figure S6: Expression profiling and functional analysis of conditioned media from nsEN and shEN HSC.

Appendix Table S1: Complete list of cytokines analyzed in the conditioned media of shEN and nsEN HSC.

Appendix Figure S1

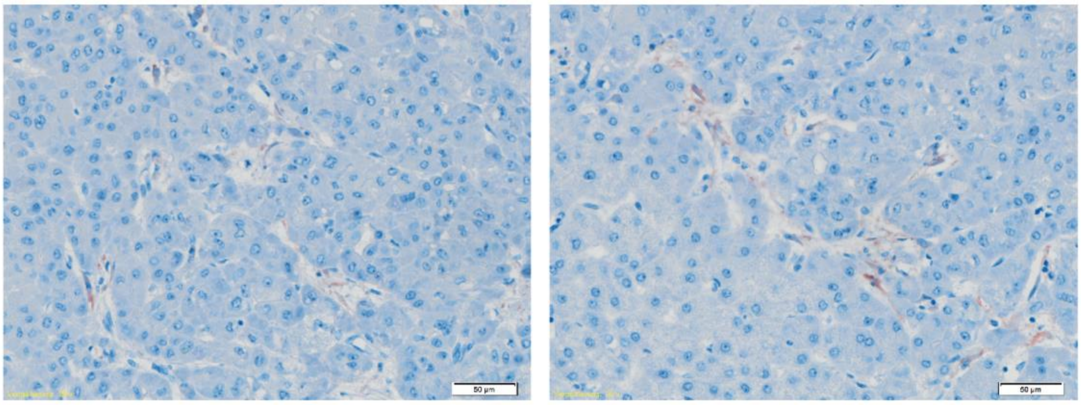

Appendix Figure S1. Endosialin expression on human dysplastic nodules (whole tissue slides). Immunohistochemical staining for Endosialin on whole tissues slides of Dysplastic nodules (200x magnification). Scale bar: as indicated.

Appendix Figure S2

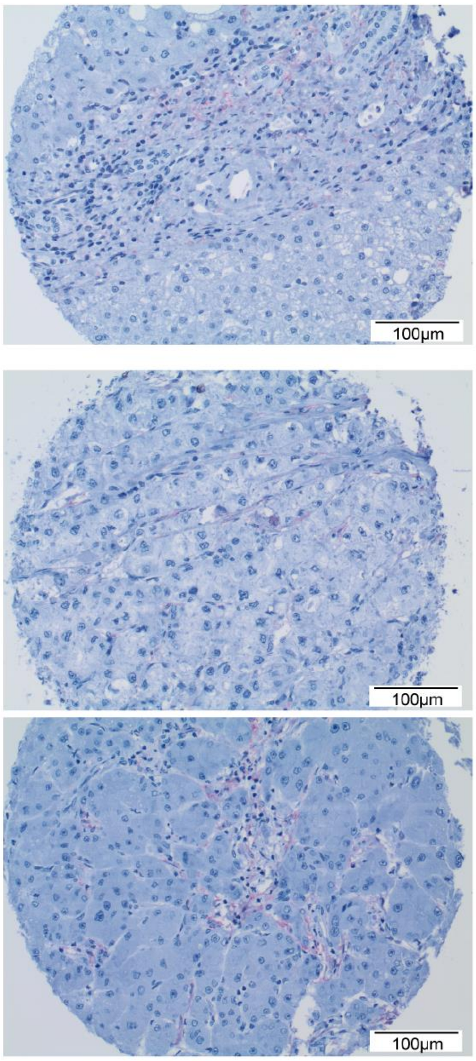

Appendix Figure S2. Heterogeneous Endosialin expression on HCC tissue microarray punches. Immunohistochemical stainings of Endosialin on 57 HCC and peritumorous liver parenchyma tissue microarray punches. Scale bars: as indicated. HCC=hepato-cellular carcinoma.

Appendix Figure S3

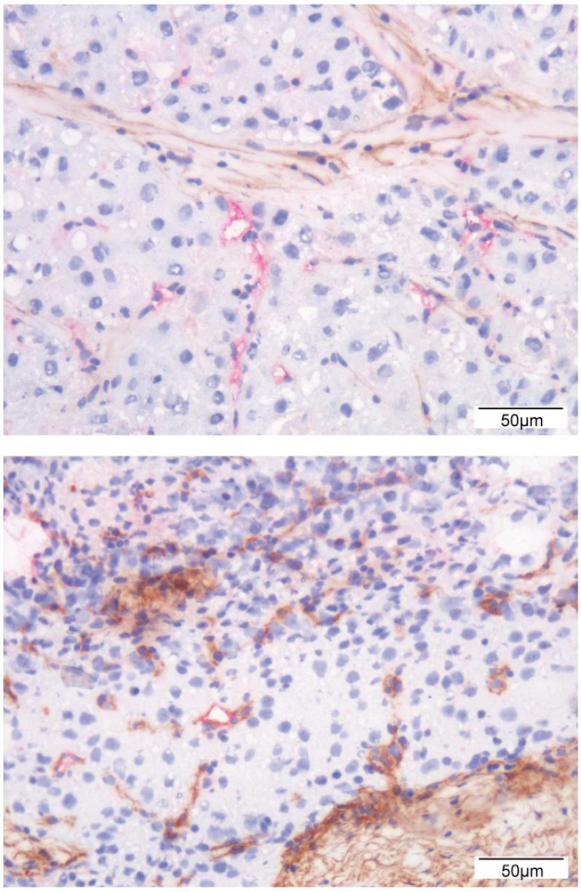

Appendix Figure S3. Co-stainings of Endosialin and CD31 on human HCC. Immunohisto-chemical double staining of Endosialin (brown) and CD31 (red) of different HCC samples (n=8). Scale bars: as indicated.

Appendix Figure S4

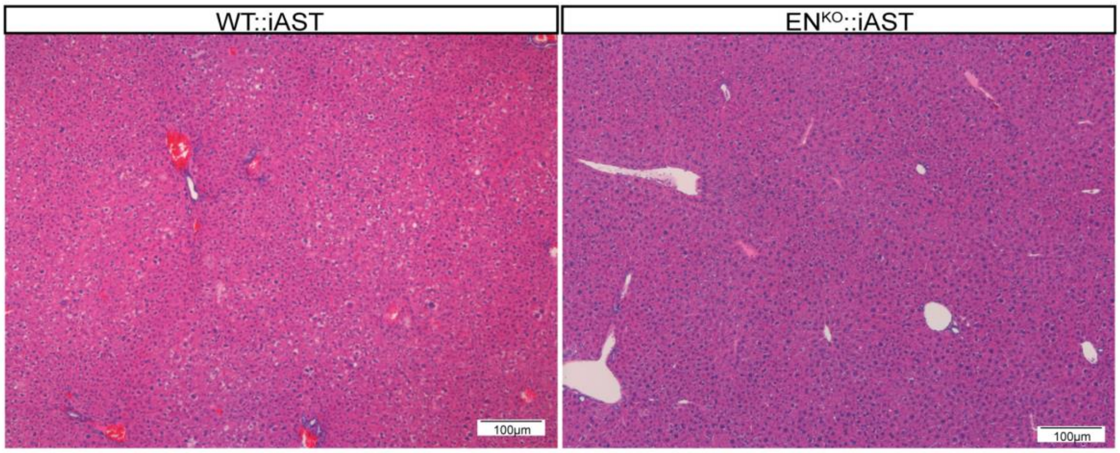

Appendix Figure S4. Immunohistochemical analysis of non-tumorigenic WT and Endosialin-deficient livers. HE stainings of WT::iAST and EN^{KO}::iAST livers in un-challenged settings do not display any overt phenotype. Scale bars: as indicated.

Appendix Figure S5

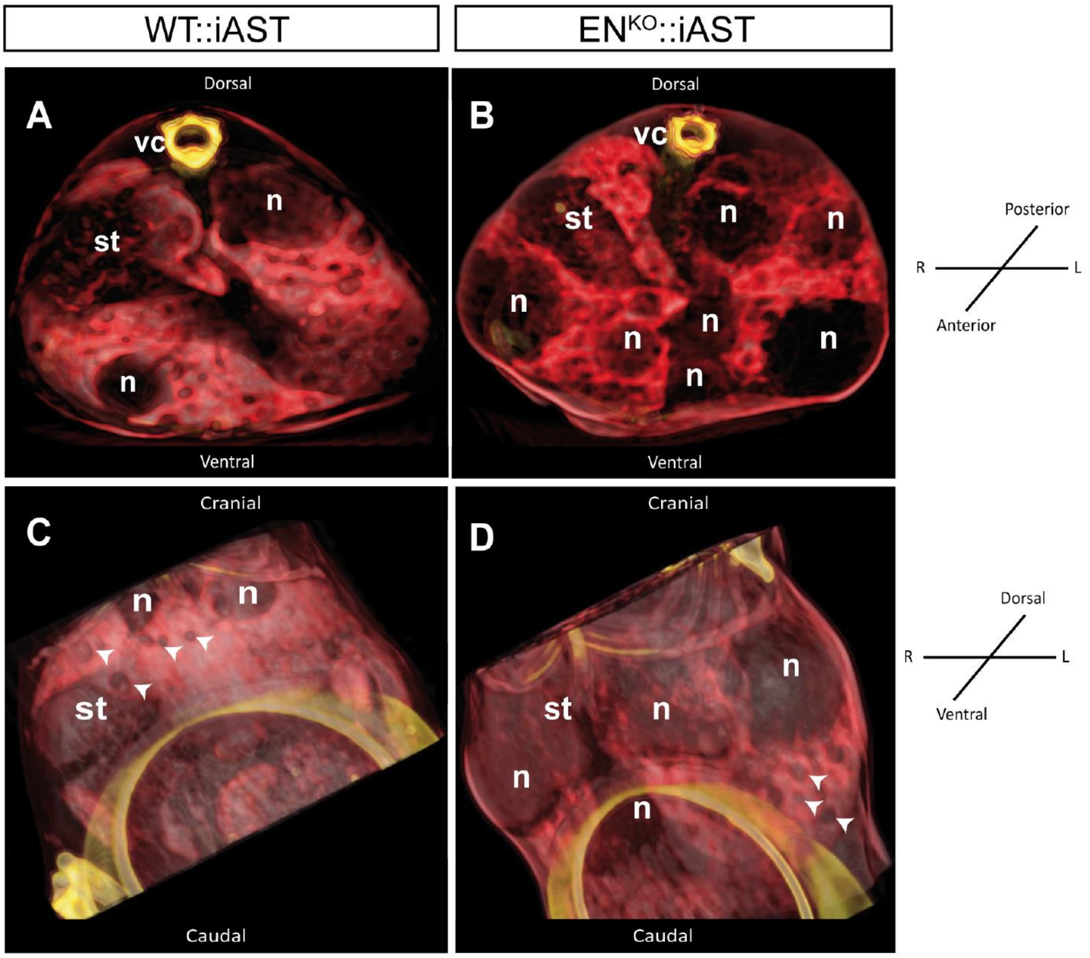

Appendix Figure S5. Computer tomographical analysis of tumorigenic WT and Endosialin-deficient mice. Computer tomography of WT::iAST (A, C,) and EN^{KO}::iAST (B,D) mice seven weeks after tumor induction as described in Figure 2. 2-dimensionally and 3-dimensionally reconstructed images. n= nodule; st=stomach; vc= vertebral cord.

Appendix Figure S6

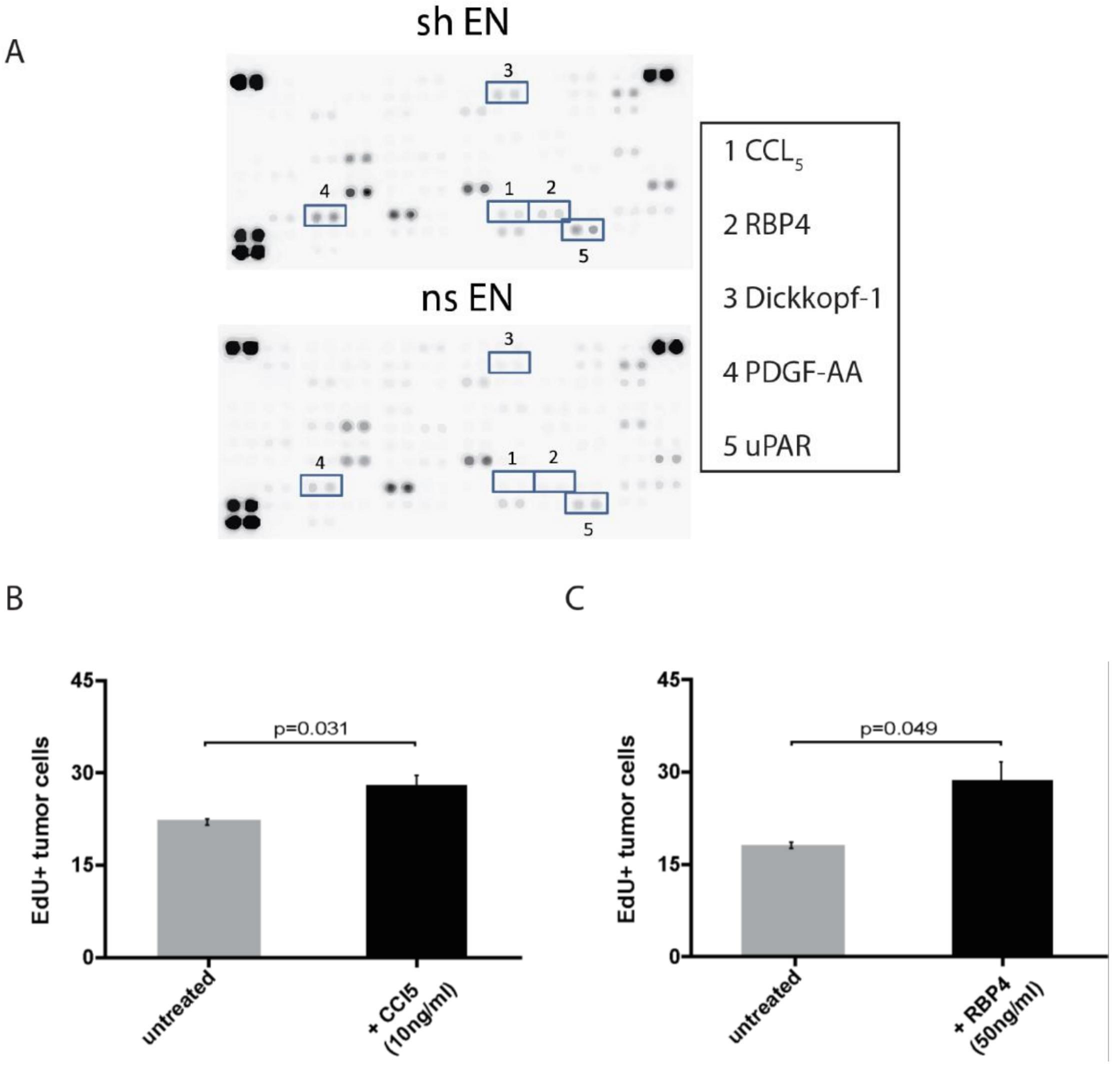

Appendix Figure S6. Expression profiling and functional analysis of conditioned media from nsEN and shEN HSC. Cytokine array of CM from nsEN and shEN HSC highlighting the top 5 differentially expressed cytokines (A). EdU proliferation assay of Huh7 tumor cells stimulated with the top 2 cytokine candidates: CCl5 (B) or RBP4 (C). n=1 with 3 technical replicates. Data are expressed as mean ± SD. Statistical analysis: Student´s t-test.

Appendix Table S1.

List of cytokines. Human cytokine array from the conditioned media of shEN and nsEN HSC.

Cytokine	nsEN	shEN	shEN/nsEN
RANTES/CCL5	2.32	14.63	6.31
RBP4	3.57	18.31	5.13
DKK-1	6.60	21.04	3.19
PDGF-AA	22.49	59.18	2.63
uPAR	22.50	54.57	2.43
CCL2/MCAF/MCP-1	58.57	132.86	2.27
Flt-3 Ligand	1.06	2.17	2.05
IL-1 R4/ST2L	1.90	3.60	1.89
Cripto-1	1.36	2.49	1.83
Osteopontin (OPN)	7.19	12.74	1.77
Complement Component C5/C5a	0.93	1.48	1.59
DPPIV/CD26/DPP4	0.59	0.94	1.59
TGFA	0.34	0.52	1.51
FGF-7	0.81	1.21	1.49
IGFBP-2	1.06	1.55	1.46
MMP-9	42.84	58.20	1.36
Endoglin/CD105	8.89	11.03	1.24
Thrombospondin-1/TSP-1	17.63	21.86	1.24
Relaxin-2/RLN2/RLXH2	2.62	3.16	1.21
MIF	99.51	118.73	1.19
CD14	6.68	7.68	1.15
LIF	1.42	1.54	1.09
EMMPRIN	42.89	46.29	1.08
Cxcl4/PF4	2.15	2.31	1.08
SHBG/ABP	5.36	5.66	1.06
TFF3/ITF	5.63	5.94	1.05
CCL7/MCP-3/MARC	1.31	1.37	1.05
GDF-15/MIC-1	3.11	3.25	1.04
CD71/TfR	3.15	3.21	1.02
G-CSF	0.66	0.68	1.02
Cystatin C	2.40	2.39	0.99
CD30/TNFRSF8	4.64	4.60	0.99
IL-13	1.23	1.18	0.96
CXCL11/I-TAC	0.71	0.68	0.96
Serpin E1	239.90	231.04	0.96
IL-15	249.04	239.28	0.95
CXCL10/IP-10	3.41	3.23	0.94
IGFBP-3	3.49	3.29	0.94
CCL3/CCL4/MIP-1a/b	1.03	0.96	-0.36
IL-10	2.55	2.34	0.92
FGF-19	16.08	14.76	0.92
GM-CSF	13.59	12.06	0.89
PDGF-AB/BB	1.16	1.03	0.88
Pentraxin-3/PTX3/TSG-14	128.18	112.91	0.88
CSF-1/M-CSF	5.49	4.81	0.88
Cxcl12/SDF-1a/PBSF	12.79	11.16	0.87
Resistin	9.56	8.32	0.87
VEGF	4.16	3.61	0.87
IL-8	71.49	61.13	0.86
IL-31	2.19	1.86	0.85
IL-23	1.06	0.89	0.84
IL-34	0.51	0.41	0.81

Cytokine	nsEN	shEN	shEN/nsEN
EGF	7.47	5.89	0.79
CRP	5.22	4.08	0.78
BDNF	4.69	3.66	0.78
Ang-1	2.66	2.04	0.77
IL-17A	24.04	18.02	0.75
IL-3	1.97	1.46	0.74
FGF basic/FGF-2	7.44	5.49	0.74
IL-11	7.10	5.21	0.73
Complement Factor D	4.12	2.98	0.72
IL-18 Bpa	1.58	1.14	0.72
CXCL5	2.96	2.11	0.71
IL-16	1.51	1.07	0.71
IL-6	9.28	6.51	0.70
Angiogenin	1.51	1.04	0.69
IL-27	4.32	2.96	0.68
IL-1b	4.13	2.81	0.68
Vitamin D BP	6.21	4.12	0.66
CXCL9/MIG	2.96	1.95	0.66
IL-19	4.27	2.64	0.62
Ang-2	8.08	4.97	0.61
IL-24	5.26	3.23	0.61
IL-1a	6.12	3.71	0.61
IL-22	7.25	4.39	0.61
IL-32a/b/g	3.02	1.82	0.60
Adiponectin	5.89	3.54	0.60
ICAM-1/CD54	3.36	2.01	0.60
IL-12 p40	2.97	1.76	0.59
TNFa	1.76	1.03	0.58
CCL17/TARC	3.11	1.79	0.58
IL-1ra	2.09	1.15	0.55
IFN-g	5.88	3.21	0.55
Lipocalin-2	5.24	2.84	0.54
BAFF/BLyS/TNFSF13B	2.74	1.48	0.54
Kallikrein 3/PSA/KLK3	5.19	2.75	0.53
IL-33	2.02	1.04	0.51
CD40 ligand/CD40L/TNFSF5/CD154/TRAP	7.67	3.93	0.51
IL-4	2.01	1.01	0.50
Fas Ligand	2.82	1.37	0.49
IL-2	2.01	0.96	0.48
IL-5	1.89	0.81	0.43
RAGE	1.03	0.41	0.40
Myeloperoxidase	3.28	1.19	0.36
CCL20/MIP-3a	0.94	0.31	0.33
Chitinase 3-like 1	2.49	0.80	0.32
Aggrecan 1	2.73	0.84	0.31
CXCL1/GRO-a	2.44	0.72	0.29
Leptin/OB	3.06	0.88	0.29
Growth hormone	1.95	0.44	0.23
HGF	2.63	0.58	0.22
CCL19/MIP-3b	0.64	0.09	0.14
