# Supplementary material for: Hepatic stellate cells limit hepatocellular carcinoma progression through the orphan receptor endosialin
Source: EMBO Mol Med. 2017 Apr 3;9(6):741–9. doi: 10.15252/emmm.201607222 (PMC5452049; doi:10.15252/emmm.201607222)
Supplement: Supplementary file 2 — Expanded View Figures PDF [file EMMM-9-741-s002.pdf]

Expanded View Figures

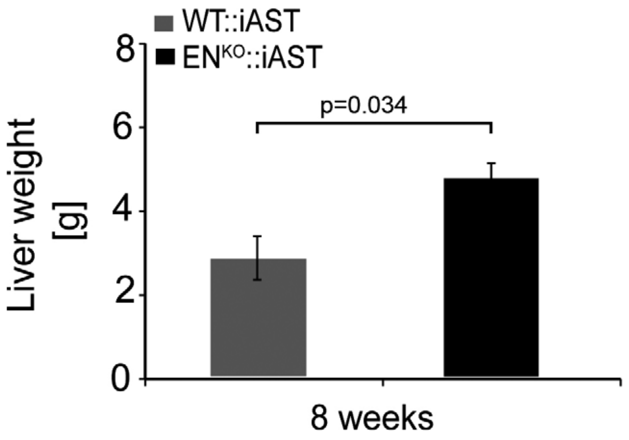

Figure EV1. Enhanced total liver weight in EN^{KO}::iAST mice.
Tumorigenesis was induced as described in Fig 2 (n = 10–12 mice per group). Total liver weight was measured 7 weeks after tumor induction. Data are expressed as mean ± SD; g = gram. Statistical analysis: Student's t-test.

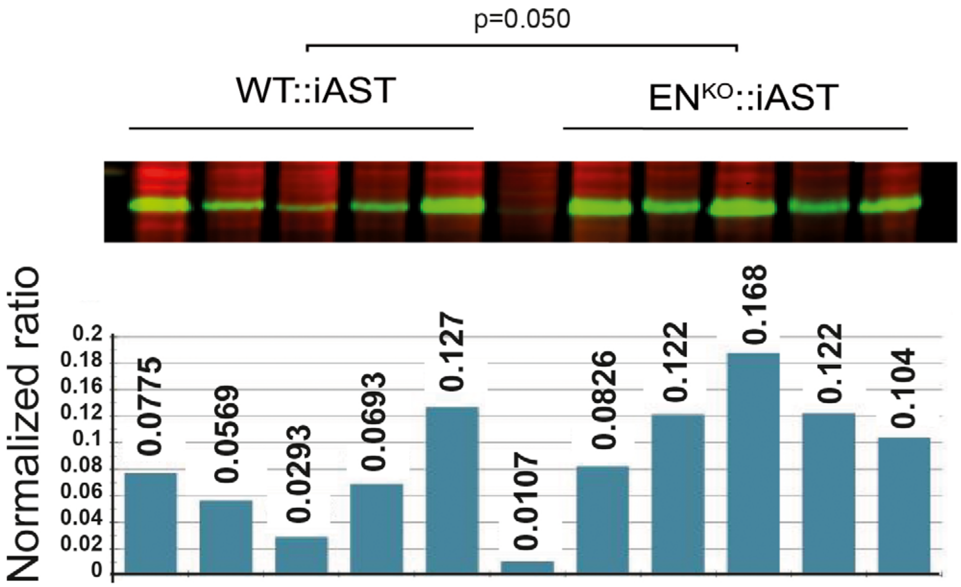

Figure EV2. Enhanced proliferation in endosialin-deficient mice.
PCNA Western blot analysis of liver lysates from WT::iAST (n = 5) and EN^{KO}::iAST (n = 5) mice taken 7 weeks after tumor induction as described in Fig 2. Quantitation of PCNA protein normalized to total protein. Statistical analysis: Student's t-test.

 © 2017 The Authors

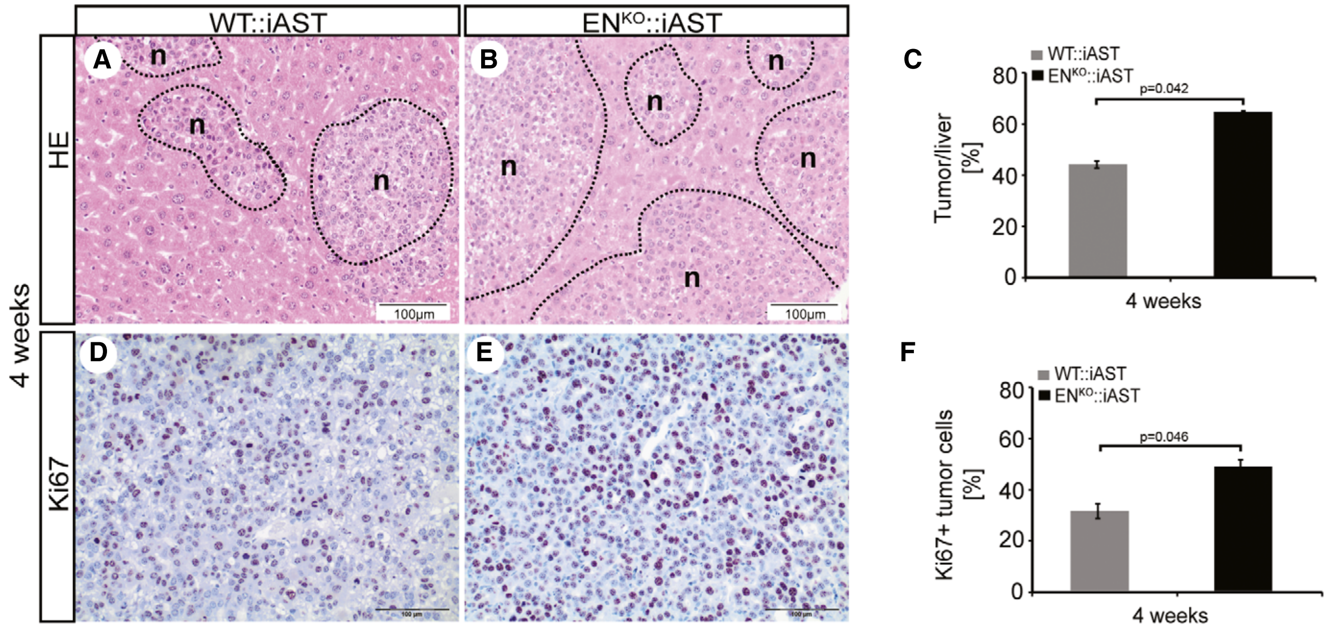

Figure EV3. Enhanced HCC tumorigenesis in endosialin-deficient mice after 4 weeks of tumor induction.

A–F HE and Ki67 staining of tumor samples from WT::iAST (A, D) and EN^{KO}:iAST (B, E) mice 4 weeks after tumor induction as described in Fig 2 (n = 5–6 mice/group). (C, F) Quantitation of tumor area/liver and Ki67 proliferation. Data are expressed as mean ± SD. Scale bars: as indicated. Statistical analysis: Student's t-test.

© 2017 The Authors
