# Supplementary figures and images for: Hepatic stellate cells limit hepatocellular carcinoma progression through the orphan receptor endosialin
Source: EMBO Mol Med. 2017 Apr 3;9(6):741–9. doi: 10.15252/emmm.201607222 (PMC5452049; doi:10.15252/emmm.201607222)

Mogler et al.: Western blot IGF2 und tubulin (Fig. 3F)

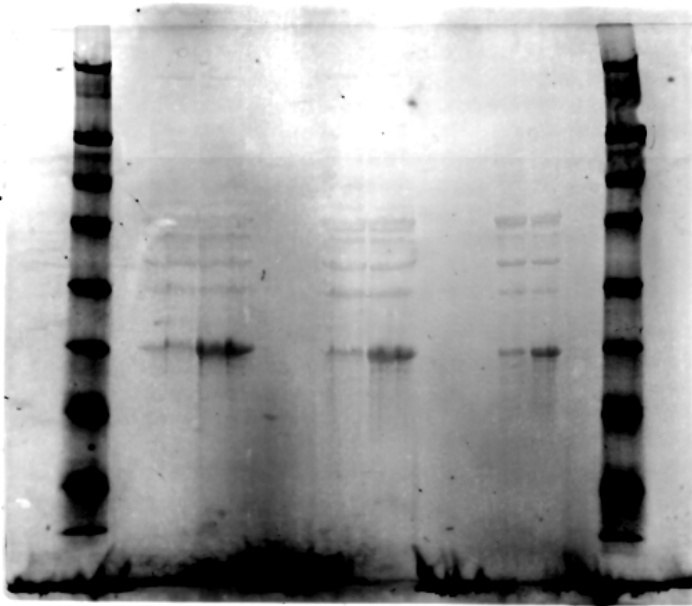

Western blot IGF2

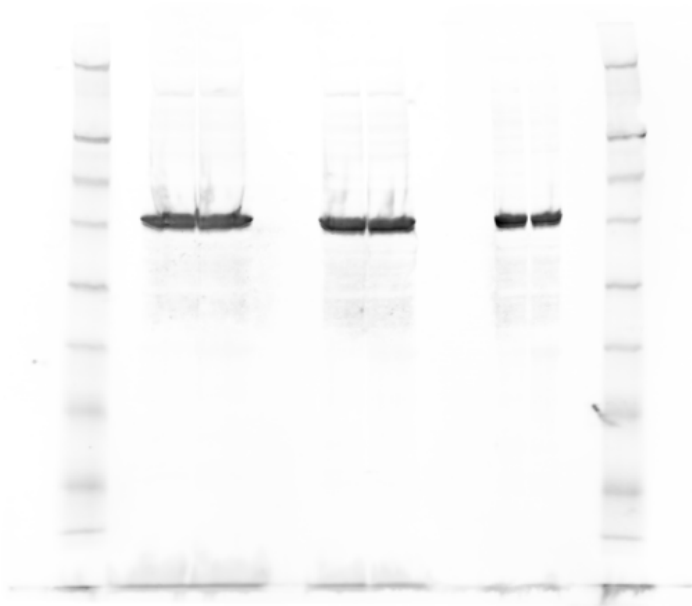

Supplement: Supplementary file 4 — Source Data for Figure 3 [file EMMM-9-741-s003.pdf]
